# “Biological Adhesion” is a Significantly Regulated Molecular Process during Long-Term Primary In Vitro Culture of Oviductal Epithelial Cells (Oecs): A Transcriptomic and Proteomic Study

**DOI:** 10.3390/ijms20143387

**Published:** 2019-07-10

**Authors:** Joanna Budna-Tukan, Agata Światły-Błaszkiewicz, Piotr Celichowski, Sandra Kałużna, Aneta Konwerska, Patrycja Sujka-Kordowska, Maurycy Jankowski, Magdalena Kulus, Michal Jeseta, Hanna Piotrowska-Kempisty, Małgorzata Józkowiak, Paweł Antosik, Dorota Bukowska, Mariusz T. Skowroński, Jan Matysiak, Michał Nowicki, Bartosz Kempisty

**Affiliations:** 1 Department of Histology and Embryology, Poznan University of Medical Sciences, 60-781 Poznan, Poland; 2 Department of Inorganic and Analytical Chemistry, Poznan University of Medical Sciences, 60-870 Poznań, Poland; 3 Department of Anatomy, Poznan University of Medical Sciences, 60-781 Poznan, Poland; 4 Veterinary Center, Nicolaus Copernicus University in Toruń, 87-100 Torun, Poland; 5 Department of Obstetrics and Gynecology, University Hospital and Masaryk University, 601 77 Brno, Czech Republic; 6 Department of Toxicology, Poznan University of Medical Sciences, 61-631 Poznan, Poland

**Keywords:** oviductal epithelial cells, biological adhesion, microarray, mass spectrometry, pig

## Abstract

Oviductal epithelial cells (OECs) actively produce stimulating and protecting factors, favoring survival and viability of gametes and early embryos. The oviduct participates in the initial reproductive events, which strongly depends on adhesion. The analysis of differential gene expression in OECs, during long-term in vitro culture, enables recognition of new molecular markers regulating several processes, including “biological adhesion”. Porcine oviducts were stained with hematoxylin and eosin, as well as with antibodies against epithelial markers. Then, OECs were long-term in vitro cultured and after 24 h, 7, 15, and 30 days of culture were subjected to transcriptomic and proteomic assays. Microarrays were employed to evaluate gene expression, with Matrix-assisted laser desorption/ionization-time of light (MALDI-TOF) mass spectrometry applied to determine the proteome. The results revealed proper morphology of the oviducts and typical epithelial structure of OECs during the culture. From the set of differentially expressed genes (DEGs), we have selected the 130 that encoded proteins detected by MALDI-TOF MS analysis. From this gene pool, 18 significantly enriched gene ontology biological processes (GO BP) terms were extracted. Among them we focused on genes belonging to “biological adhesion” GO BP. It is suggested that increased expression of studied genes can be attributed to the process of intensive secretion of substances that exhibit favorable influence on oviductal environment, which prime gametes adhesion and viability, fertilization, and early embryo journey.

## 1. Introduction

The oviduct is a cylindrical structure extending between the ovary and the uterus. It is organized into three segments (the infundibulum, the ampulla, and the isthmus) and four histological layers (mucosa, submucosa, muscularis, and serosa). The innermost layer of mucosa presents variable character, depending on the region. In the infundibulum, it forms finger-like folds, which become highly developed and branched in the ampulla, to finally decrease and adopt simplified structure in the isthmus [1]. Regardless of the region, mucosa is lined with simple columnar epithelium, comprised of ciliated and non-ciliated cells, presenting secretory and motor function. i.e., oviductal epithelial cells (OECs) [2,3]. During the estrus cycle, under hormonal changes, proportions of OEC types and their morphology undergo substantial alterations, with prevalence of ciliated cells in the follicular phase and secretory cells in the luteal phase [1].

The abovementioned morphology and location of the oviduct justify its participation in the initial reproductive events. Many of them involve formation or disruption of tight connections between somatic cells, gametes, embryos, and reproductive organs, and thus it strongly depends on adhesion abilities. The process of biological adhesion is indispensable for intercellular communication, signal transduction, proliferation, or apoptosis [4]. Cellular connections include: cytoskeleton-involving adherens junctions (AJs), formed by integrins, cadherins, and desmosomes [5]; selective tight junctions, controlling the molecular, and ionic flow, made up of claudins and occludins [6]; and lastly, gap junctions, based on connexins, allowing small substance flow between adjacent cells [7].

Oocytes, in cumulus-oocyte complexes (COCs), form tight junction connections with cumulus cells (CCs), allowing the flow of stimulating factors necessary for oocyte maturation [7]. In the majority of mammalian species, oocyte maturation takes place in the ovarian follicle, and is followed by disruption of follicular wall and ovulation. As a result, compact structures of the COCs loosen. Ovulated COCs transiently adhere to OECs of oviductal ampulla before they start to flow towards uterus. The oviduct promotes the oocyte’s journey with the use of three mechanisms: movement of the fimbriae of the infundibulum, beating movement of OEC cilia, and peristaltic contractions of the muscularis layer [8]. Simultaneously, counter-current movement of the sperm occurs. However, before this may happen the oviduct modifies the spermatozoa, after their passage from the cervix through utero-tubal junction, making them capable of fertilization. Some sperm migrate directly to the ampulla, while the majority adhere to the OECs, forming reservoir pending for capacitation at the time of ovulation [9]. Transient adhesion of COCs to OECs is most likely the signal indicating the occurrence of ovulation, triggering sperm hyperactivation and release [10]. This passage is crucial for successful fertilization and ensures that only a low number of selected spermatozoa reaches the ampullar-isthmic junction where fertilization takes place [11]. The latter is highly dependent on E-cadherin assured by intercellular adhesion [12]. Following fertilization, ciliary action eases the transport of the zygote towards uterus [8].

The precise mechanism of gamete and embryo interactions with OECs has not yet been described. Although intrauterine implantation is a definite sign of pregnancy, it is known that communication processes begin much earlier. Large preovulatory follicles, characterized by intense hormonal production, were shown to alter OEC gene expression differently than small preovulatory follicles, presumably creating an optimal oviductal environment for the future embryo [13]. Moreover, non-fertilized oocytes stay in the oviduct longer, while fertilized ones are directly transferred to the uterus [14]. The presence of embryos in the oviduct of pigs, cows, and horses was shown to change the transcriptome of the OECs, especially involving genes related to immune response, thus protecting the embryo from rejection [15]. All this points to a conclusion that OECs actively produce stimulating, protecting, and growth factors, favoring survival and viability of gametes and early embryos [16,17,18].

Thus, many co-culture systems are implemented, including those employing the porcine model, to mimic in vivo COC-OEC interactions and to improve the efficiency of COCs’ in vitro maturation. Among them, co-culture with OECs significantly improved nuclear maturation of canine oocytes [19], blastocyst formation frequency, total cell number, and subsequent embryo development in pigs [20] and humans [21]. It is defined that co-cultures with OECs are beneficial, since they enrich culture medium with growth factors and cytokines, as well as work as medium detoxifiers and developmental block inhibitors [20].

The biggest clinical opportunity associated with OECs is the possible application of their co-cultures in improvement of the in vitro fertilization (IVF) outcomes. Hence, there is a large need to analyze the changes of their function outside of the physiological conditions. The long-term in vitro cultures of OECs could bring their specific ex vivo features, as well as the ability to secrete stimulating factors, into light. The analysis of differential gene and protein expression in OECs during vitro long-term cultivation represents a good and stable model for recognition of molecular markers of several processes, including “biological adhesion”, which could indicate long-term viability of those cells for potential clinical applications. 

Therefore, the main aim of this study is to analyze the changes in the transcriptomic and proteomic profile of cultured OECs, in relation to that detected closely to the point of collection (24 h of culture). The authors presume that the changes in gene and protein expression will, to some extent, mirror the effects of the ex vivo culture conditions on the physiological properties of the OECs. The knowledge gained could then be used to better understand the processes that might also occur during OEC-containing co-cultures. This, in turn, could serve to improve the methodology of OEC-gamete/embryo co-cultures, which ultimately serves in maximizing the positive outcomes of the IVF procedures. 

## 2. Results

### 2.1. Histological Evaluation

**Figure 1:**
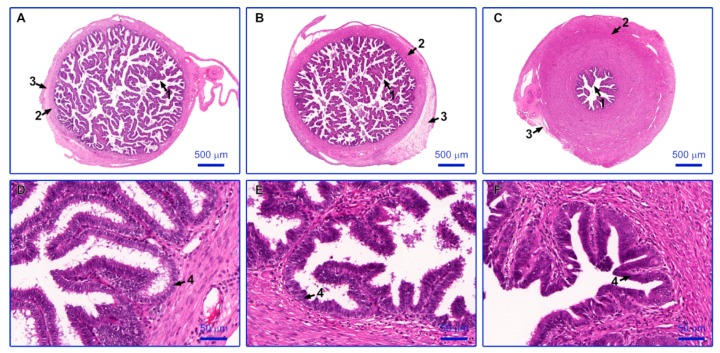
Representative sections of porcine oviducts stained with hematoxylin and eosin. (**A**) the infundibulum, (**B**) the ampulla, (**C**) the isthmus, (**D**–**F**) epithelium lining the mucosal folds, in the infundibulum, the ampulla and the isthmus, respectively. Arrows: 1: mucosal folds, 2: muscle layer, 3: serosa, and 4: simple columnar ciliated epithelium.

Observation of paraffin sections stained with the hematoxylin and eosin (H+E) method allowed to assess the morphological structure of the various parts of the oviduct. Each of the analyzed sections showed normal histological structure. Particular segments of oviduct vary in the thickness of the muscle layer and the length and number of mucosal folds. The infundibulum contains long, finger-like mucosal folds and a thin muscle layer. In the ampulla, mucosa forms numerous, branched folds, while the muscle layer is thicker compared to the infundibulum. The isthmus is characterized by a thick layer of smooth muscles distributed in circular and longitudinal layers, while the folds of the mucosa are poorly developed. In each segment of the oviduct, the mucosal folds are lined with the simple columnar ciliated epithelium. Non-prominent cilia indicate anestrous period of the ovarian cycle. The photographed results were presented in Figure 1.

### 2.2. Oviductal Epithelial Cell (OEC) Culture

**Figure 2:**
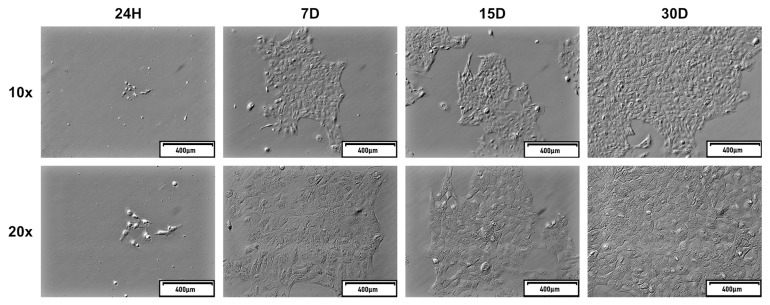
The representative images of oviductal epithelial cells (OECs) primary long-term in vitro culture taken after 24 h, 7, 15, and 30 days of culture using an inverted microscope with relief phase contrast.

The OECs subjected to long-term cell culture did not significantly change their morphology during its period. The pictures, depicting cells in all of the timeframes from which the material for molecular analyses was collected, present the same epithelial-like shape throughout the culture time (Figure 2).

### 2.3. Microarray Expression Analysis

The investigation of OEC transcriptome following 24 h, 7, 15, and 30 days of long-term in vitro culture yielded 14,789 porcine transcripts. From this group, after seven days of culture, 1146 genes met the criteria of significantly changed expression; 733 of them were up-regulated and 413 were down-regulated. After 15 days of culture, 938 genes were up-regulated and 562 were down-regulated, giving 1500 differentially expressed genes in total. Finally, after 30 days of culture, from the 1970 genes that significantly altered their transcript levels, 1444 were up-regulated and 526 were down-regulated.

### 2.4. nanoLC-MALDI-TOF/TOF MS/MS Analysis

**Figure 3:**
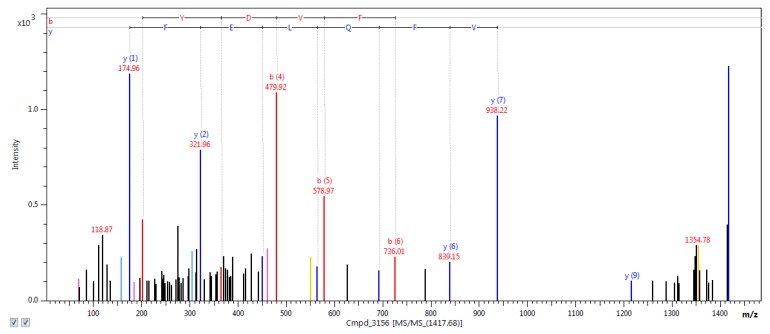
The representative example of the mass spectrum of *m*/*z* 1417.6834, identified as one of the fascin peptides.

The nano liquid chromatography-matrix-assisted laser desorption/ionization-time of light tandem mass spectrometry (nanoLC-MALDI-TOF/TOF MS/MS) analysis allowed for the identification of the total number of 3240 unique tryptic peptides, which correspond to 389 proteins. The representative example of the mass spectrum (*m*/*z* 1417.6834), which was identified as one of the fascin peptides, is shown in Figure 3. In the database used, there was no possibility to restrict the search to the *Sus scrofa* species. Hence, the “Other mammals” database was searched, which yielded some proteins assigned to species other than pig. The list of those proteins was presented in Supplementary Table S1. As they were detected in porcine samples, it can be assumed that these proteins are homologues of those found in *Sus scrofa*. Moreover, some additional searches were made including “Homo sapiens” and “Rodentia” datasets, in order to complete obtained molecular pattern. Results were presented in Tables S2 and S3.

### 2.5. Genes and Proteins of Interest

**Figure 4:**
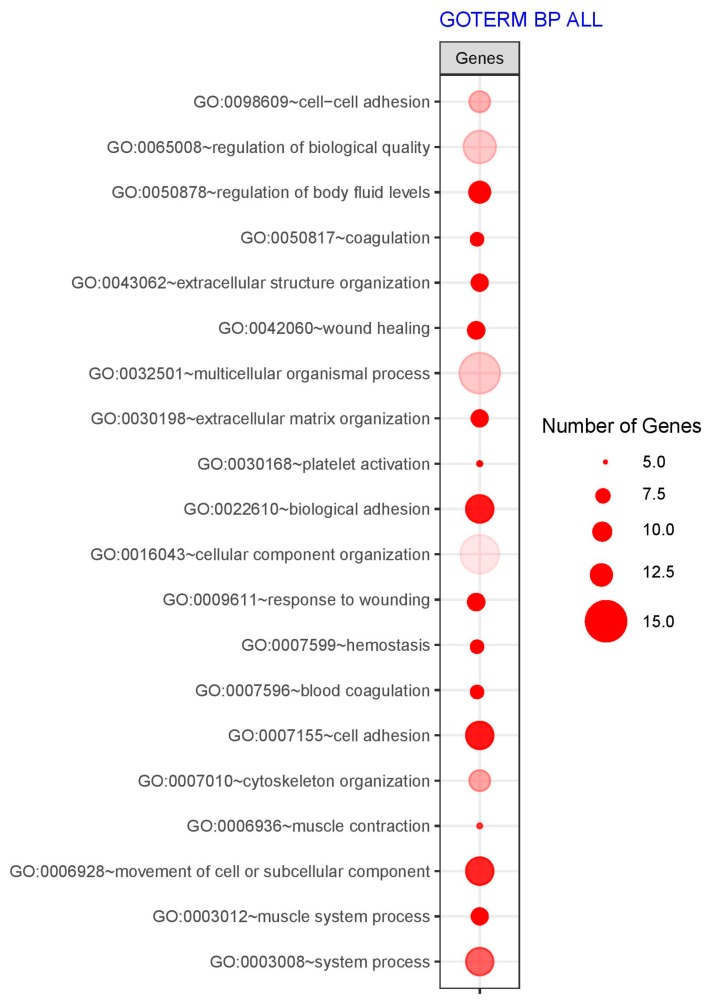
The plot showing the list of enriched gene ontology biological processes (GO BP) terms. The size of the circle corresponds to the number of genes. The color intensity indicates the fold enrichment of the terms.

From the differently regulated genes, we chose the 130 porcine analogs of genes encoding proteins detected by MALDI-TOF analysis. From this pool, genes belonging to the 20 significantly enriched Gene Ontology Biological Processes (GO BP) terms were provided by the DAVID software. The list of enriched GO BP terms and the amount of differently expressed genes were shown in Figure 4. 

**Figure 5:**
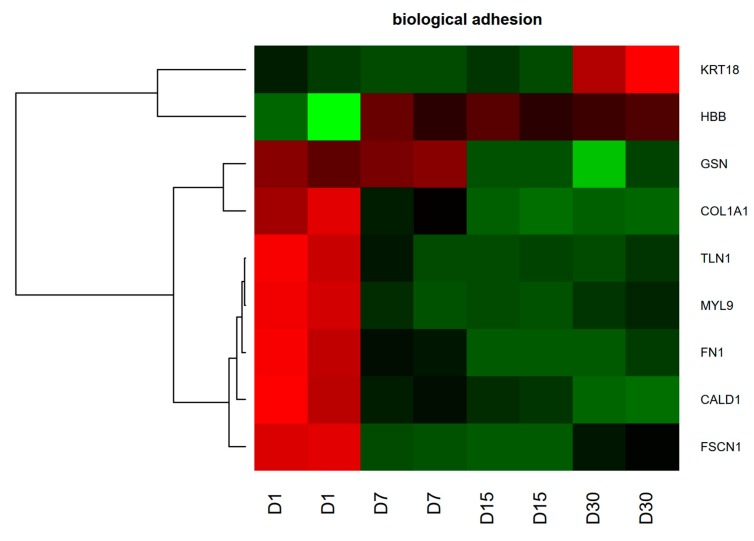
Heatmap representation of differentially expressed genes belonging to “biological adhesion” GO BP term. Microarray signal intensity values for each gene were adapted to Row Z-Score scale ranging from −2 (the lowest expression) to +2 (the highest expression) and reflected by red and green colors, respectively.

In this manuscript, we focused on the genes that belong to “biological adhesion” GO BP. The hierarchical clustering of selected genes was carried out and result was presented as a heatmap (Figure 5). 

### 2.6. RT-qPCR Analysis of Genes of Interest

**Figure 6:**
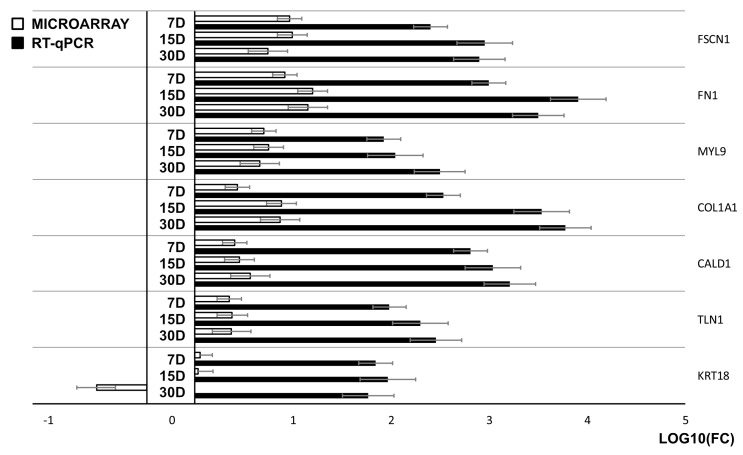
The results of the microarray and real-time quantitative polymerase chain reaction (RT-qPCR) analyses presented in a form of a bar graph.

The results of the microarray analysis were validated using the real-time quantitative polymerase chain reaction (RT-qPCR) method. Most of the patterns of expression changes were consistent between both of the methods. *Keratin 18* (*KRT18*) was an exception, showing opposite direction of change on day seven of primary culture. Furthermore, the scale of the detected alterations was bigger in RT-qPCR than in microarrays. As this pattern is consistent across all of the analyzed genes, it can be assumed that it results from the difference in sensitivity of both methods. All of the data obtained was presented and compared on a bar graph (Figure 6).

### 2.7. Western Blot Analysis of Proteins of Interest

**Figure 7:**
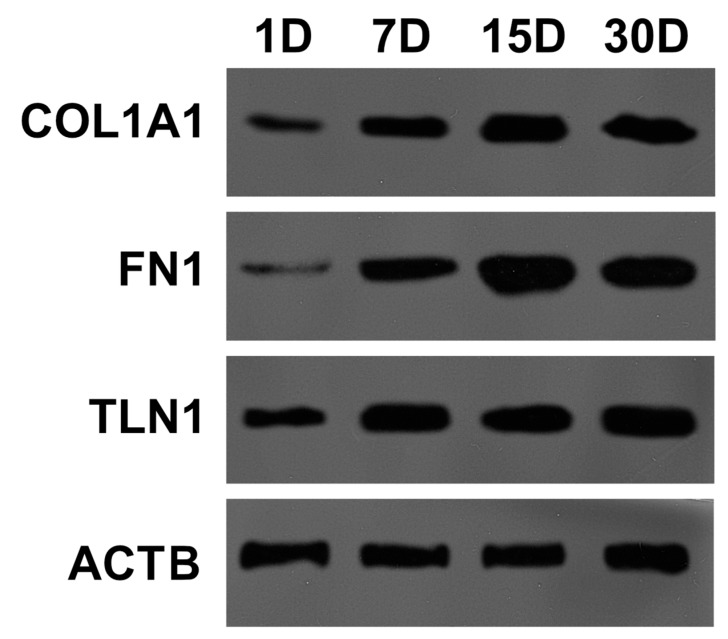
The changes in Collagen Type I Alpha 1 Chain (COL1A1), Fibronectin 1 (FN1), Talin 1 (TLN1), and Actin Beta (ACTB) proteins expression in 24 h, 7, 15, and 30 days of OEC culture.

The results of MALDI-TOF analysis were validated using the Western blot method. It was performed to indicate changes in Collagen Type I Alpha 1 Chain (COL1A1), Fibronectin 1 (FN1), and Talin 1 (TLN1) protein levels during OEC primary long-term in vitro culture (Figure 7) as genes encoding them presented the most substantial changes in RNA expression. The protein analysis showed an increased expression of COL1A1, FN1, and TLN1 proteins in 7, 15, and 30 days of OEC culture by ~300%. However, an increase in FN1 protein level was more pronounced in 15 and 30 days of culture (~500%) as compared to those in 24 h.

## 3. Discussion

Previous transcriptomic studies on bovine OECs revealed groups of genes exhibiting altered expression dependent on the phase of estrous cycle [22] and specific for either the ampulla or the isthmus [23]. Among others, the most prominent changes in OECs isolated from the ampulla were related to genes associated with cell migration and motility, ciliary movement, and beat frequency. In the isthmus, similar findings indicated genes associated with: nitrogen, lipid, cholesterol, and nucleotide synthesis, cell cycle, vesicle-mediated transport, and apoptosis [24]. In our study, we only discuss genes that were differentially expressed during 30 days of OEC culture, protein products of which were also found in cells on the last day of culture. Presence of protein on the 30th day of culture was not always reflected by the change of its gene expression during the course of cultivation, suggesting the protein has stable expression during culture periods. Selected genes and proteins were found robustly in several ontology groups related to crucial cellular processes. From the group of those belonging to the “cellular adhesion” GO BP, the genes which attracted our attention were *Keratin 18* (*KRT18*), *Gelsolin* (*GSN*), *Fibronectin 1* (*FN1*), *Collagen Type I Alpha 1 Chain (COL1A1*), *Talin 1* (*TLN1*), *Fascin Actin Bundling Protein 1* (*FASCN1*), *Myosin Light Chain 1* (*MYL9*), *Hemoglobin Subunit Beta* (*CALD1*), and *Hemoglobin Subunit Beta* (*HBB*).

The process of adhesion plays an important role in reproductive events associated with both gametes and embryos. During their maturation, the oocytes form tight gap junctions connections with cumulus cells (CCs), enabling achievement of the MII stage of the female gamete [7]. Additionally, recent studies show that adhesion has a great impact on post-ovulatory journey of the oocyte through the oviduct. It was found in cows that interactions between COCs and OECs start immediately after the COCs’ entry to the oviductal ampulla [8], promoting hyperactivation of sperm bound in the isthmus and its counter-current movement towards ampulla [10]. Specifics of the events facilitating transport of the oocyte, as well as its interactions with the OECs, remain unclear. Nevertheless, in vitro co-cultures of COCs with ampullar and isthmic OECs was found to be beneficial, increasing the time of zona pellucida digestion and monospermic penetration rate [25]. 

Sperm interactions with the OECs are certainly better described [26]. The mechanism of sperm detention in the caudal isthmus is based on their adhesion to OECs, and is assured by binding of Arg-Gly-Asp amino acid sequence on the spermatozoa with integrins on the OECs [27]. After capacitation and hyperactivation, sperm loses its binding affinity, presumably due to modification of surface proteins and physical disruption of connections [3]. It is assumed that this interaction affects changes in oviductal genes’ expression, mainly related to ciliary motility [28], muscularis contractions [29], intercellular transport, and communication [30]. Moreover, adhesion of male and female gametes to the epithelial cells lining the oviduct promotes alterations in their proteome [31]. Additionally, OECs-provided maintenance of embryonal growth was associated with synthesis of various growth factors [25]. Therefore, it is assumed that OECs are programmed to support fertilization and further embryo development [32].

Keratin 18 is a protein regarded as a marker of epithelial cells. Its high expression is found in mammalian single-layered epithelia, such as single columnar epithelium lining the oviduct. Its main role, together with keratin 8, is the formation of intermediate filaments (IF), creating structural framework of epithelial cells [33]. Although keratins are mainly known for their ability to strengthen and stabilize cells, they can indirectly influence intercellular adhesion. The latter can be facilitated by clusters of desmosomal cadherins, supported by IF-made desmoplakins and plakophilins [34]. However, it is worth keeping in mind that the confluence of cultured cells, and thus number of cell junctions, increases along the culture time [35], which may prove to be an alternative explanation for increased KRT18 expression. In microarray results, an exception was noted on day 30, when KRT18 expression dropped. However, the RT-qPCR validation did not confirm this result. Thus, increased expression of KRT18 and the presence of keratin 18 during the culture may reflect not only in vitro induced changes of the OEC but also their enhanced adhesiveness, possibly promoting interactions with gametes. 

The oviduct promotes gamete and early embryo transport [3]. The dominant role in this passage is attributed to action of cilia that cover the simple columnar epithelium lining the oviduct [36]. One of the proteins involved in ciliogenesis is gelsolin. This calcium-regulated protein is responsible for assembly and disassembly of actin filaments. Its ability to bind to “plus” ends of actin monomers and filaments prevents monomer exchange and three-dimensional structure formation. Experiments have shown that small interfering ribonucleic acid (siRNA)-mediated depletion of GSN causes decreased count of ciliated cells, indicating that actin filaments severing plays a role in ciliogenesis [37]. However, it is widely known that cells grown in in vitro monolayers are prone to dedifferentiation and lose their morphological features, including cilia [38]. Thus, we speculate that the increase in GSN expression in our culture was associated with other processes. It is known that Gelsolin is related to many other pathways in mammals, especially adhesion. Analysis of extracellular vesicles isolated from the oviductal fluid (oEVs) revealed transcripts and proteins belonging to few GO terms, including cell adhesion. Gelsolin was one of the members of the group of proteins most abundant across the estrous cycle, with profound impact on sperm–oviduct interaction and early embryonic development [39]. It was found in uterine epithelial cells that actin binding proteins, including Gelsolin, are involved in regulation of endometrial skeleton, as well as in embryo attachment to the epithelium via focal adhesion kinase interaction [40].

Adhesion is a process strictly linked to membrane-associated proteins called integrins, which can bind to neighboring cell membrane or elements of extracellular matrix (ECM) [41]. They are known receptors of an adhesive glycoprotein: fibronectin 1 [42]. So far, reported functions of FN1 in mammalian reproduction are related to sperm-oocyte binding, implantation and placentation, while none of them attributed to the oviductal journey [43,44]. In silico protein interaction studies, related to oocytes maturation, fertilization, and embryo growth in sheep, showed that FN1, together with other proteins, favors cell growth in in vitro conditions [45]. Goossens et al. observed in cattle that FN1 expression level increased from the morula to the blastocyst stage, even before embryo implantation. It raises the possibility that embryonic FN1 matrix gains adhesive properties to keep cells together, as well as to enable following migration of embryonal cell layers [46]. Although the developing zygote travels passively towards the uterus, where maternal recognition of pregnancy takes place, it can exert signals “informing” about its presence and modifying expression of specific genes in oviductal cells. As the result, oviduct secretes factors with highly beneficial influence on embryo development and/or decreases activity of the immune system [47,48,49,50]. 

Another ECM component, collagen type I alpha 1 chain(COL1A1), is an element of fibril-forming collagen, a crucial component of connective tissue, abundantly present in bone, tendons, and dermis. Its high expression was also found in other tissues and organs, including endometrium and placenta [51,52]. However, there were no reports, until know, about its presence in oviducts. It is possible that the synthesis of FN1 and COL1A1 extracellular proteins can influence the microenvironment of oviducts in some manner. However, results of our experiments, conducted without hormonal supplementation, cannot be referred to changes occurring during the pregnancy and are more likely attributed to increasing confluency of cultured cells.

The involvement of focal adhesion, linking cellular integrins with ECM, has been already noted in reproductive events [40,46,53,54,55]. Talin 1 (TLN1) couples integrins to actin cytoskeleton, playing part in integrin-mediated events [56]. Compact structure of COCs is guaranteed by focal adhesion complexes composed, among others, by TLN1. Kawashima et al. revealed in mice that, in response to LH surge, COCs undergo the process of expansion and CC movement caused by disruption of focal adhesion proteins, such as TLN1 [57]. Subsequently, events triggered by fertilization in mammals are integrin-dependent and thus, indirectly TLN1-dependent. As the result, oocytes become activated through cytoskeletal structures [58]. Furthermore, the forming blastocyst, to complete the implantation process, interacts with uterine epithelial cells through integrins. It was presented in mice that downregulation of TLN1 resulted in impaired adhesion and implantation rate, while its disruption inhibited formation of focal adhesion and subsequently led to embryo death [59]. This outcome emphasizes the crucial role of cytoskeletal proteins and their ligands for proper adhesion and implantation of blastocyst [60]. Lack of information about the role of TLN1 in the mammalian oviduct directed our attention towards epithelial cultures. Experiments showed that different culture conditions and coatings influence TLN1 expression [61,62]. Increased expression of TLN1 in our culture, similarly like FN1 and COL1A1, can reflect high confluency and stable cultivation of porcine OECs, even on the 30th day of in vitro culture. However, we cannot exclude the possibility that these genes exert functions stimulating oviductal environment, as well as benefit gamete and embryo passage. 

Apart from the abovementioned genes and their products, we also selected a group with known function in adhesion process but without proven role in reproduction, including: fascin actin bundling protein 1 (FASCN1) [63,64,65,66,67], myosin light chain 1 (MYL9) [68], caldesmon 1 (CALD1) [69], and hemoglobin subunit beta (HBB) [70]. Further examination may explain their yet unrevealed potential in reproduction.

In summary, high expression of some of the adhesion-related genes and proteins can be explained by their role during reproduction in processes such as oocyte or spermatozoa binding to the epithelium lining the oviduct, while for some of them there are no reports describing mentioned relations. OECs in vitro culture is stable and widely used model, that brings some of the physiological features of those cells into light. However, despite numerous advantages, cells cultured ex vivo, deprived of their usual microenvironment, may undergo transcriptomic and proteomic changes that reflect in their phenotype. Thus, direct translation of in vitro culture results to pathways related to reproduction in vivo is not a simple task and requires a large amount of further studies. However, beneficial effect of co-culture of gametes and embryos with OECs has been already proven. Therefore, we can speculate that increased expression of genes and proteins may reflect the extent of physiological properties of the OECs that is maintained during the process of long-term in vitro culture. This in turn can be attributed to intensive secretion of substances with favorable influence on oviductal environment, which prime gamete adhesion and viability, fertilization, and early embryo journey. Hence, functional tests are needed to precisely designate their share in mimicking in vivo conditions.

## 4. Material and Methods

Data availability: All raw data generated during the research and used to obtain results presented in this manuscript is available from the corresponding author on a reasonable request.

### 4.1. Tissue Collection

Oviducts were collected from 45 crossbred gilts from a commercial herd. The animals at the age of approximately nine months, which displayed two regular estrous cycles, were slaughtered after reaching the anestrus phase of the cycle. Collected tissues were immediately transported to the laboratory, kept in an isolated container. The research related to animal use has been complied with all the relevant national regulations and institutional policies for the care and use of animals. Bioethical Committee approval no. 32/2012 from 1 June 2012.

### 4.2. Histological Evaluation

Shortly after the collection of tissues, oviducts from 10 gilts were sectioned to separate the infundibulum, ampulla and isthmus regions. Subsequently, tissues were fixed for 48 h in Bouin’s solution, dehydrated, embedded in paraffin blocks and cut with a rotary microtome into sections of 3–4 µm. Obtained paraffin sections were stained using a hematoxylin and eosin (H + E) staining technique, following a previously described protocol [71]. Analysis of all sections was performed under a light microscope, with pictures taken using high-resolution scanning technique (Olympus BX61VS microscope scanner, Olympus, Tokyo, Japan).

### 4.3. Primary Long-Term Culture of OECs

The method used for OEC harvesting and culture was based on the available literature sources [72,73]. Collected oviducts were washed twice in Dulbecco’s phosphate buffered saline (PBS) and sectioned longitudinally. OECs were gently scratched with the use of sterile surgical blades and subsequently enzymatically digested for 1 h at 37 °C with 1mg/mL collagenase I (Sigma Aldrich, St. Louis, MO, USA) in Dulbecco’s modified Eagle’s medium (DMEM; Sigma Aldrich). Cell suspension was filtered through 40 µm pore size strainer and centrifuged for 10 min at 200× *g*. After rinsing with PBS, OECs were incubated for another 10 min at 37 °C with 0.5% Trypsin/EDTA (Sigma Aldrich), filtered and centrifuged as described above, and suspended in DMEM supplemented with 10% FCS, 100 U/mL penicillin, 100 µg/mL streptomycin, and 1 µg/mL amphotericin B. The cells were cultured for up to 30 days at 37 °C, in a humidified atmosphere of 5% CO_2_. The culture medium was changed every three days. Once OECs reached confluency of around 80%, they were passaged to another culture dish at the seeding density of 2 × 10^4^ cells/cm^2^. Throughout the time of the culture, pictures of cell morphology were taken using an inverted microscope employing relief phase contrast (Olympus IX73, Olympus).

### 4.4. RNA Isolation from Oviductal Epithelial Cells (OECs)

OECs were collected, pooled and suspended in TRI Reagent (Sigma Aldrich) after 24 h, 7, 15, and 30 days of culture. Total mRNA was isolated from samples using RNeasy MinElute cleanup Kit (Qiagen, Hilden, Germany). The amount, purity, integrity and quality of total mRNA were determined as described previously [74]. Each mRNA sample was diluted to 100 ng/µL concentration.

### 4.5. Microarray Expression Analysis and Statistics

The 100 ng of mRNA from each pooled sample was transferred to succeeding microarray procedure described in details in our recent paper [75]. Briefly, primary cDNA was obtained and amplified (Ambion^®^ WT Expression Kit, Ambion Inc., Thermo Fisher Scientific, Waltham, MA, USA). Then, cDNA was labelled with biotin and fragmented (Affymetrix GeneChip^®^ WT Terminal Labeling and Hybridization, Affymetrix, Santa Clara, CA, USA). Resulting fragments were hybridized to the microarray strips (Affymetrix^®^ Porcine Gene 1.1 ST Array Strip, Affymetrix), which were afterwards washed, stained according to the manufacturer’s protocol (Affymetrix GeneAtlas Fluidics Station, Affymetrix), scanned (Imaging Station of the GeneAtlas System, Affymetrix) and analyzed (Affymetrix GeneAtlasTM Operating Software, Affymetrix), resulting in generation of raw CEL files containing all of the data.

Downstream analyses were performed, and graphs were obtained using Bioconductor and R programming languages, as well as the Robust Multiarray Averaging (RMA) algorithm. Statistical significance of analyzed genes was determined with the use of moderated t-statistics from the empirical Bayes method, with obtained p-value corrected for multiple comparisons using Benjamini and Hochberg’s false discovery rate. Genes regarded as differentially expressed presented p-value beneath 0.05 and expression higher than 2-fold.

### 4.6. Protein Digestion

Cells harvested from culture flasks were centrifuged and rinsed twice with 50mM ammonium bicarbonate solution to remove residues of the culture medium. Subsequently, the sample was suspended in 1 mL of 50% methanol in a solution of 0.1% trifluoroacetic acid (TFA) in water, mixed for 1 min and incubated at −20 °C for 5 min. The procedure of mixing and incubation was repeated three times. Then, the sample was sonicated for 15 min and centrifuged. The supernatant was divided into three tubes and subjected to the protocol for in-solution tryptic digestion, according to modified Pierce Kit procedure (Thermo Scientific, USA). After overnight digestion, samples were purified and concentrated using C18 ZipTip micropipette tips (Millipore, Burlington, MA, USA) according to the protocol provided by the manufacturer.

### 4.7. nanoLC-MALDI-TOF/TOF MS/MS Analysis

Three purified samples were fractionated using nano-liquid chromatography (nanoLC): EASY-nano LC II connected with Proteineer-fc II fraction collector (Bruker Daltonics, Billerica, MA, USA). Detailed conditions of the performed separation, as well as specifications of LC columns were described in our previous research [76]. 384 fractions were obtained, with each automatically mixed with HCCA matrix solution and spotted onto MALDI target plate (MTP AnchorChip 384, Bruker Daltonics, Billerica, MA, USA). Subsequently, MALDI-TOF/TOF MS/MS analysis was performed using the UltrafleXtreme instrument (Bruker Daltonics, Billerica, MA, USA). Typical settings of the apparatus for MS and MS/MS mode were presented in our previous study [76]. MS spectra were externally calibrated with the use of peptide calibration mixture in the 0.7–3.5 kDa mass range (Bruker Daltonics). WARP-LC 1.3 platform (Bruker Daltonics, Billerica, MA, USA) was used to select precursor ions with signal-to-noise threshold above 5 for MS/MS analysis. The following platforms and software were used for analysis, data acquisition and evaluation: WARP LC 1.3, ProteinScape 3.1, FlexControl 3.4, and FlexAnalysis 3.4. (Bruker Daltonics, Billerica, MA, USA).

Protein search was conducted using the National Center for Biotechnology Information (NCBI) database with Mascot 2.4.1 search engine (Matrix Science, London, UK) using the following parameters: trypsin digestion, 1 missed cleavage, peptide mass tolerance: 25 ppm; MS/MS fragment mass tolerance: 0.7 Da; peptide charge: 1+; monoisotopic mass; and carbamidomethylation of cysteine as fixed modification. The searches were restricted to “Other mammals”. Additional searches were made including “Homo sapiens” and “Rodentia” datasets. Only proteins with at least two unique peptides, score above 80 and false discovery rate below 1% were included. Results obtained from three analysis were combined into one list of the proteins generated by the ProteinScape software.

### 4.8. Selection of Genes of Interest

Since not every gene encodes a protein, changes in gene expression are not always correlated with protein changes. To select differentially expressed genes accompanied by their protein products, NCBI eFetch tool was used to match obtained genes with their corresponding protein products detected in MALDI-TOF MS analysis. Subsequently, pig analogues of these genes were found, with only the differentially expressed genes from these pairs used in following analysis. 

Selected genes were analyzed according to an already published sequence [77]. Briefly, the genes were uploaded to the Database for Annotation, Visualization, and Integrated Discovery software (DAVID, Laboratory of Immunopathogenesis and Bioinformatics, Frederick, MD, USA) [78] to extract enriched GO BPs. Only GO BPs containing at least five genes and with a p-value lower than 0.05 were chosen. 

### 4.9. RT-qPCR Validation

The relative changes in gene expression results obtained from the microarray analysis was validated with the use of real-time quantitative polymerase chain reaction (RT-qPCR). Primers specific to genes of interest were designed using the Primer3 software. Exon-exon design method was used as an additional measure to avoid amplification of genomic DNA fragments. The primers were also designed to allow amplification of all of the transcription variants of genes of interest found in the Ensembl database. The sequences of primers used were presented in Table 1. The reaction mix included cDNA samples, forward and reverse primers, and SYBR Green I as a detection dye. Negative controls were processes with primers replaced with highly purified water to ensure lack of non-specific amplification. HPRT, ACTB, and GAPDH were used as housekeeping genes. Water samples were also included as blanks, to ensure its purity and eliminate background noise. The reactions were processed, each in three technical repetitions, in the LightCycler 96 Real-Time PCR system (Roche Diagnostics, Mannheim, Germany). The specificity of amplification was estimated based on the dissociation curves provided by the software provided by the equipment manufacturer. The transcript levels, as well as the fold changes in expression were determined using the 2^-ΔΔC_T_ method.

### 4.10. Sodium Dodecyl Sulphate-Polyacrylamide Gel Electrophoresis (SDS-PAGE) and Western Blot Analysis

The expression levels of several proteins of interest were validated using Western blot analysis. Three proteins were selected based on the most substantial changes in their RNA expression. Total protein of OECs was isolated with RIPA lysis buffer. A total of 15 µg protein was re-suspended in sample buffer and separated on 8% Tris-glycine gel with the aid of SDS-PAGE. The proteins were transferred to PVDF membrane, which was blocked with 5% milk in Tris buffered saline/Tween. Immunodetection was performed with rabbit polyclonal anti-COL1A1 Ab (ab34710), rabbit polyclonal anti-Fibronectin 1 (FN1) Ab (ab2413), and rabbit polyclonal anti-Talin 1 (TLN1) Ab (ab71333) followed by incubation with goat anti-rabbit HRP Ab (ab205718). The membranes were also incubated with anti-actin (ACTB) HRP Ab (sc-1616) to equalize protein loading of the lanes. SuperSignal West Femto maximum sensitivity substrate Pierce Biotechnology Inc. (Rockford, IL, USA) was used to reveal bands.

## Figures and Tables

**Table 1:** Sequences of primers used for the real-time quantitative polymerase chain reaction (RT-qPCR). analysis.

Name	Forward Primer (5’–3’)	Reverse Primer (5’–3’)
*FN1*	TGAGCCTGAAGAGACCTGCT	CAGCTCCAATGCAGGTACAG
*KRT18*	GGGCTCAGATCTTTGCAAGT	GTCTCATACTTGACTCTGAAGTCATC
*GSN*	AGAAACAGATCTGGAGAATCGAA	CGCCCTGCCAGTTGTAGAT
*COL1A1*	GGTCTCCCAGGTCCTAAGG	GCTAGGACCAGTTTCACCCT
*TLN1*	AGGCCAGAAAGAGTGTGACA	CCGTTCTTGGCATTTTGGGA
*FSCN1*	CGCAGGTCAACATCTACAGC	CTCGAGAGTGTAGCCTGTGG
*MYL9*	GGCCTTCAACATGATCGACC	GCTTCTCCCCAAACATGGTG
*CALD1*	CACAAGCTCAAACACACCGA	TCAGCTCCTCCAGTTCCAAG
*HBB*	GTGACGGCCTGAAACATCTC	CTGGCCCACAAGTACCACTA

## Supplementary Materials

The following are available online at https://www.mdpi.com/1422-0067/20/14/3387/s1, Table S1. The list of 389 proteins identified by the nanoLC-MALDI-TOF/TOF MS/MS analysis. Table S2. The list of 220 proteins identified by the nanoLC-MALDI-TOF/TOF MS/MS analysis using “Homo sapiens” dataset. Table S3. The list of 225 proteins identified by the nanoLC-MALDI-TOF/TOF MS/MS analysis using “Rodentia” dataset.
